# Comprehensive Characterization of Linalool-HP-β-Cyclodextrin Inclusion Complexes

**DOI:** 10.3390/molecules25215069

**Published:** 2020-11-01

**Authors:** María Isabel Rodríguez-López, María Teresa Mercader-Ros, Carmen Lucas-Abellán, José Antonio Pellicer, Alfonso Pérez-Garrido, Horacio Pérez-Sánchez, María Josefa Yáñez-Gascón, José Antonio Gabaldón, Estrella Núñez-Delicado

**Affiliations:** 1 Molecular Recognition and Encapsulation Research Group (REM), Health Sciences Department, Universidad Católica de Murcia (UCAM), Campus de los Jerónimos 135, Guadalupe, 30107 Murcia, Spain; mirodriguez@ucam.edu (M.I.R.-L.); mtmercader@ucam.edu (M.T.M.-R.); clucas@ucam.edu (C.L.-A.); japellicer@ucam.edu (J.A.P.); mjgascon@ucam.edu (M.J.Y.-G.); jagabaldon@ucam.edu (J.A.G.); 2 Structural Bioinformatics and High Performance Computing Group (BIO-HPC), Computer Engineering Department, Universidad Católica de Murcia (UCAM), Campus de los Jerónimos 135, Guadalupe, 30107 Murcia, Spain; aperez@ucam.edu (A.P.-G.); hperez@ucam.edu (H.P.-S.)

**Keywords:** linalool, HP-β-cyclodextrins, solid complexes, chemical characterization, molecular modeling

## Abstract

The objective of the present study is to obtain linalool- cyclodextrin (CDs) solid complexes for possible applications in the food industry. For this purpose, a detailed study of linalool complexation was carried out at different pH values, to optimize the type of CDs and reaction medium that support the highest quantity of encapsulated linalool. Once demonstrated the ability of hydroxypropyl-β-cyclodextrin (HP-β-CDs), to form inclusion complexes with linalool (K_C_ = 921 ± 21 L mol^−1^) and given their greater complexation efficacy (6.788) at neutral pH, HP-β-CDs were selected to produce solid inclusion complexes by using two different energy sources, ultrasounds and microwave irradiation, subsequently spraying the solutions obtained in the Spray Dryer. To provide scientific solidity to the experimental results, the complexes obtained were characterized by using different instrumental techniques in order to confirm the inclusion of linalool in the HP-β-CDs hydrophobic cavity. The linalool solid complexes obtained were characterized by using ^1^H nuclear magnetic resonance (^1^H-NMR) and 2D nuclear magnetic resonance (ROSEY), differential scanning calorimetry, thermogravimetry and Fourier transform infrared spectrometry. Moreover, the structure of the complex obtained were also characterized by molecular modeling.

## 1. Introduction

Nowadays, the demand for natural compounds in the food industry has increased due to the need to obtain more biodegradable preservatives [1,2]. Essential oils (EOs) have been known since the Middle Ages due to their antiseptic, therapeutic properties and their intense aroma, arousing great interest in food preservation. The difference in their organoleptic profiles is conditioned by their chemical composition and richness, characteristics that determine their antioxidant and antimicrobial properties [3], which have aroused the interest of large companies related to human and animal nutrition, pharmacy and cosmetics [4]. The antimicrobial action of the EOs is due to their ability to penetrate through the bacterial membranes into the cell, causing the inhibition of their vital functions [5,6,7,8]. EOs are very complex natural mixtures characterized by two or three main components in fairly high concentrations (20–60%) [9].

Linalool (3,7-dimethyl-1,6-octadien-3-ol) is an alcoholic monoterpene with a floral scent present at high concentration in different plants such as *Lavandula* sp. [10], being the majority component of coriander essential oil (68%) [11,12], in *Ocinum Basilicum* L. (56.7–60.6%) [13], and in the *Lauraceae* family from which the EOs of laurel, cinnamon and rosewood are obtained [14]. In addition to the natural sources, linalool can be obtained by organic synthesis from α, β-pinene or other terpenes (geraniol, nerol, myrcene), or via the 2-methyl-2-hepten-6-one route [15]. The problem with synthetic linalool is the presence of traces of dihydrolinalool and dehydrolinalool [16], and even chlorinated impurities that provide a metallic nuance to the linalool aroma [17].

Linalool is a colorless liquid, relatively soluble in water (1590 mg L^−1^ at 25 °C) and highly soluble in different organic solvents. This tertiary terpene alcohol has a chiral center at C3 carbon, thus appearing two enantiomeric forms in nature: (+)-Linalool and (−)-linalool [18]. It was approved by the Food and Drug Administration (FDA) as GRAS (generally recognized as safe), thus authorizing its use as a spice or food additive [19]. In addition, the Joint Expert Committee (FAO/WHO) on Food Additives (JECFA) established an allowable daily intake (ADI) of 0–0.5 mg kg^−1^ of body weight/day for linalool [20]. Therefore, its use is very widespread in different sectors, being used as a flavoring agent in the food industry, as a fragrance in the perfume and cosmetic industry and in different drug formulations in the pharmaceutical industry [21].

Being the majority component of many EOs, numerous works describe linalool contribution to their biological activity as antimicrobials, anti-leishmaniosis, anti-inflammatories and antioxidants [22,23,24,25,26]. In relation to the antimicrobial activity and due to its aliphatic alcohol structure, it has been noted the ability to bind to different molecular structures such as proteins or glycoproteins; therefore, linalool has a high affinity for cell membranes and a high potential to penetrate through walls, facilitating the leakage of cytoplasmic material out of the cell [27].

One of the main drawbacks related to linalool is the capability to cause skin irritations by skin absorption, contact eyes and inhalation, coughing, pain, choking and breathing difficulties [28]. This problem could be solved by promoting its microencapsulation in cyclodextrins (CDs), since this technique has been used successfully in the food industry to improve the stability and viability of EOs [9,28]. CDs are cyclic sugars able to form inclusion complexes with a large number of molecules, since they have a hydrophobic cavity that can trap some EOs components, and a hydrophilic surface that makes them soluble in water [9,28]. This unique property of CDs is governed by an equilibrium constant (K_C_) and provides many applications of interest in the pharmaceutical, food or analytical chemistry industries. In previous studies, linalool and camphor from lavender EOs were encapsulated in β-CDs to control the release of fragrances [29]. Ciobanu et al. [29], obtained stable complexes capable of reducing the volatility of aromatic compounds.

Therefore, the present study aimed to standardize a basic methodology for the encapsulation process of linalool, as model compound, with different CDs types, as well as a detailed characterization of the solid complexes formed by ^1^H-NMR, Fourier transform infrared spectroscopy (FT-IR) and differential scanning calorimetry (DSC), in order to evidence the inclusion of linalool into the CDs hydrophobic cavity. In addition, the strength of interactions, geometry, structural aspects and conformation of inclusion complexes formed were also explored by using scanning electron microscopy (SEM) and molecular docking.

## 2. Results and Discussion

### 2.1. Complexation of Linalool with CDs

To standardize an encapsulation procedure of linalool with CDs, first of all, it was made a solubility study of linalool in the presence of different types of CDs (α-, β-and HP-β-CDs) at different pH values (3.5, 5.5, 6.5, 7, 8.5) and at 25 °C.

**Figure 1:**
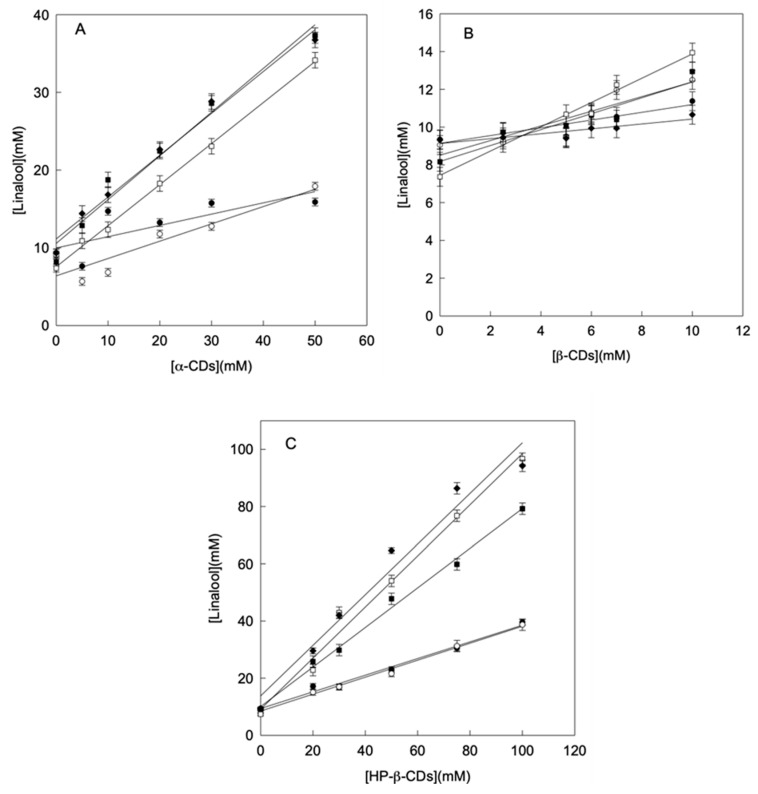
Phase solubility diagrams of linalool with α-CDs (A), β-CDs (B) and HP-β-CDs (C) at pH 3.5 (●), pH 5.5 (○), pH 6.5 (■), pH 7.0 (□) and pH 8.5 (♦). Values represent means of triplicate determination.

The concentration of linalool increased linearly with the concentration of CDs (phase solubility diagrams Type AL), indicating that the stoichiometry of the complexes formed was 1:1 in all cases (Figure 1). Derived from the phase solubility diagrams, using the Equation (1) and, in order to compare the stability of the complexes formed, the complexation constant (K_C_) between linalool and different types of CDs were calculated.

As observed in Table 1, the K_C_ values obtained increased when the pH approaches neutrality (7.0) for all CDs studied (Table 1). This result agrees with the fact that the solubility (S_0_) of linalool varies with the medium pH, being more soluble at acidic or alkaline pH (S_0_ = 9.34 mmol L^−1^) than at neutral pH 7.0 (S_0_ = 7.37 mmol L^−1^) (Table 1). It must be taken into account that linalool has a hydroxyl group at the C3 chiral carbon, which implies that the molecule behaves as a weak acid (pKa = 18.46), so its dissociation degree will be conditioned by the pH of the medium, and therefore, its solubility. The main factors limiting complex formation are the relationship between the size of internal cavity of the CDs and the host molecule, and the polarity of the host molecule. So, the protonation of linalool and ultimately its solubility is decisive in the complexation process and the stability of the complexes formed.

As can be seen in the results presented in Table 1, independently of the CDs used, the highest K_C_ value was obtained for the complexes formed at pH 7.0 (148 ± 12 L mol^−1^ for α-, 241 ± 26 L mol^−1^ for β- and 921 ± 21 L mol^−1^ for HP-β-CDs), which differs significantly from the Kc values obtained at either acidic or alkaline pHs. These results were similar than those obtained for thymol complexation, which showed the highest Kc values at neutral pH as well as linalool [9].

Despite this improvement at neutral pH, in general, the K_C_ values were low, probably conditioned by the structure and instability of linalool, since it is isomerized easily giving rise to primary alcohols (geraniol and nerol), even forming cycles, not favoring its inclusion in the hydrophobic CDs cavity. It is important to note that linalool is characterized by its chemical instability, since it is capable of oxidizing, reducing and polymerizing easily when interacts with CDs in acidic and alkaline conditions. It can suffer intramolecular transformations that can modify its structure and, consequently, modify its ability to be included in the hydrophobic cavity of CDs.

When compared K_C_ values obtained for native and modified β-CDs, the results were 241 ± 26 L mol^−1^ for β- and 921 ± 21 L mol^−1^ for HP-β-CDs at neutral pH (Table 1). The differences observed were related with the change of H atoms by hydroxypropyl groups in the case of HP-β-CDs favoring the entrapment of linalool. The K_C_ value obtained for HP-β-CDs (4-fold than that obtained for β-CDs), indicated the formation of a more stable complex due to the intensity of hydrophobic and Van der Waals interactions involved. In addition, the presence of hydroxypropyl groups is enough to break the hydrogen bridges close to the CDs cavity, making the entrance of linalool molecules more accessible to the apolar cavity. These results agree with those obtained for resveratrol [30], kaempferol and *p*-coumaric acid that present cyclic structures, or citral with a non-cyclic structure [31,32].

In summary, the stability of the soluble complexes formed between linalool and CDs depends both on the medium pH and the CDs type, being the optimal conditions pH 7.0 and HP-β-CDs.

In order to complete the solubility study, not only the stability of the complexes formed (K_C_) was analyzed, but also the complexation efficiency (CE) between linalool and CDs was calculated according to Equation (2). For 1:1 stoichiometry complexes the CE is independent of the aqueous solubility of the encapsulated compound. The comparison of CE values gives more information than comparing K_C_ values when study different types of CDs for the complexation of the same compound. As can be seen in Table 1, HP-β-CDs showed the highest CE values at all pHs studied (0.41 for pH 3.5, 0.443 for pH 5.5, 1.836 for pH 6.5, 6.788 for pH 7.0 and 5.215 for pH 8.5) (Table 1). In the case of pHs 6.5, 7.0 and 8.5, the values obtained above 1 indicated that the quantity of CDs forming complexes with linalool where higher than free CDs in solution. Similar results were previously described for eugenol with HP-β-CDs (2.81), ferulic acid with HP-β-CDs and RAMEB (1.43 and 1.79 respectively), and coumaric acid with HP-β-CDs, RAMEB and CRYSMEB (2.30, 2.57, 1.89 respectively) [33]. The rest of CDs studied, α- and β-CDs, presented CE values below 1 at most of studied pHs, indicating that most did not increase linalool solubility (Table 1).

Moreover, CE was also used to calculate the linalool:CDs molar ratio (MR) by using Equation (3), which can be correlated to the expected increase in formulation bulk. As in the case of CE, the highest value for MR, was also obtained for HP-β-CDs at pHs 6.5, 7.0 and 8.5 (1:1 in all cases) (Table 1). This 1:1 value indicated that almost all CDs in solution were forming soluble complexes with linalool. These results agree with those obtained for eugenol and coumaric acid, in which the highest EC values coincide with an MR of 1:1 [33]. In summary, taking into account K_C_, CE and MR (linalool:CDs) values, HP-β-CDs at pH 7.0 are the best conditions to increase the aqueous solubility of linalool, forming the most stable and soluble complexes.

### 2.2. Obtaining Solid Inclusion Complexes Linalool-HP-β-CDs

**Figure 2:**
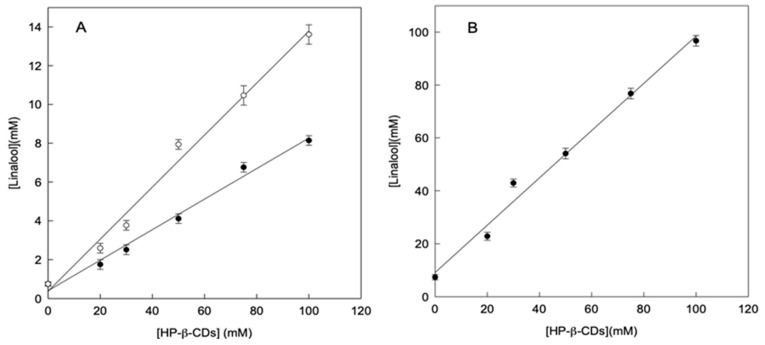
Phase solubility diagrams of linalool with HP-β-CDs a pH 7.0 by using microwave as energy source 24h MWI (○) and 48h MWI (●) (**A**), by using ultrasounds as energy source (**B**). Values represent means of triplicate determination.

In order to facilitate the handling and preservation, the formation of linalool complexes in solid state was also studied. As described previously, linalool complexes could be made by using different energy sources to reach equilibrium: microwave irradiation (MWI) or ultrasound (US). Figure 2 shows the solubility profiles of linalool in the presence of HP-β-CDs a pH 7.0, by reaching the equilibrium using both methods. As can be seen in Figure 2A, the solutions treatment with MWI (24 h MWI or 48 h MWI) to reach the equilibrium, much less increased the linalool solubility than the application of US (Figure 2B).

When the increase in solubility of linalool was compared through the preparation of complexes by using MWI or US, as an energy source, at a volume of 100 mL, it was found to be 18.2 and 13.1-fold, respectively, thus achieving a higher increase in solubility of linalool by MWI. This result is due to the fact that the S_0_ in the case of MWI (0.75 mmol L^−1^) is lower than that obtained by ultrasound (7.37 mmol L^−1^). In the case of MWI the phenomenon of molecular heating may not be enough to cause the linalool oil micro drops disaggregation. However, in the case of US, the molecular movement favors the drops disaggregation.

Therefore, the contact surface is reduced and consequently its aqueous solubility, as shown in Figure 2A. In any case, in order to obtain an aqueous solution with high concentration of linalool, the use of US should be selected as appropriate method. As can be seen in Figure 2, by using 100 mM of HP-β-CDs, the linalool concentration solved by using MWI irradiation was 13.61 mM, whereas in the case of US it was 96.72 mM (7-fold higher).

**Figure 3:**
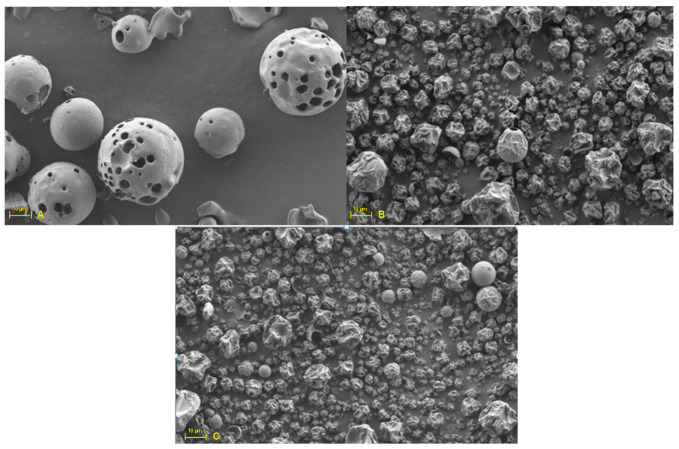
Microphotographs of HP-β-CDs (**A**), MWI complexes (**B**) and US complexes (**C**).

Soluble complexes prepared with different concentrations of HP-β-CDs were subjected to an atomization process (spray drying) to obtain solid complexes [34,35]. The shape of the particles obtained after the atomization process can be influenced by different factors such as the solid content in the solution, the inlet temperature, the spraying speed and the air nozzle of the equipment, which affect to the evaporation rate [36]. The wrinkled shape of the particles obtained for the linalool-HP-β-CDs solid complexes (Figure 3), was due to the fact that the evaporation rate on the surface of the droplets was faster than the rate at which the dissolved components were diffuse towards the center, to form a crust around the droplets [37].

As shown in Figure 3B,C, the external surfaces showed continuous walls without cracks, which significantly influence the loss of volatile compounds. This fact has also been described for oregano essential oil [38], bay leaf infusions [39] and thymol [9].

**Figure 4:**
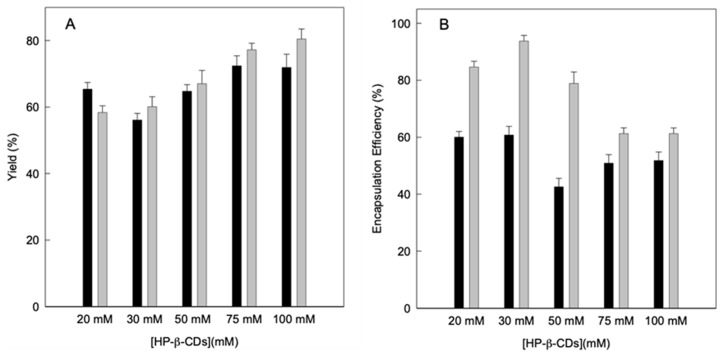
(**A**) Drying process yield (g kg^−1^) and (**B**) encapsulation efficiency of linalool (g kg^−1^) of linalool-HP-β-CDs complexes prepared by US (grey bars) and MWI (black bars). Values represent means of triplicate determination.

Subsequently, the performance of the drying process was calculated using Equations (4) and (5), preparing the complexes using different energy sources to achieve balance: MWI or US, without taking into account the dust that remains inside the equipment, since it is considered a negligible amount. As can be seen in Figure 4A, the yield is higher than 56% in all cases, so it can be considered such a good performance for both methods. However, it is evident that by increasing the concentration of HP-β-CDs a higher yield is obtained for both MWI and US, reaching the maximum yield at 100 mM for the US (80%). If compare the yield for the complexes obtained by using MWI and US at 100 mM HP-β-CDs, values of 71.9 and 80.5% were obtained, respectively (Figure 4A). Similar results were obtained for thymol complexes obtained by using the same energy sources as linalool [9]. Furthermore, it must be taken into account that the volume introduced into the equipment were small (a total volume of 100 mL), therefore losses can always occur, since on a laboratory scale it is difficult to achieve yields greater than 80% [40].

Therefore, the determination of the losses of linalool due to the drying treatment was analyzed and the concentration of linalool in the solid complexes after spraying was evaluated (Figure 4B).

These results were used to calculate the encapsulation efficiency of the complexes obtained by using both energy sources, based on the theoretical amount of linalool in the initial solution and its relationship with the amount of linalool present in the solid complexes at the end of the process. The encapsulation efficiency ranged from 42.6 to 60.8 g/100 g for the MWI and from 61.3 to 93.7 g/100 g for the US (Figure 4B).

These results can be justified by the fact that CDs form both inclusion and non-inclusion complexes, and the complexes form soluble aggregates capable of also solubilizing linalool molecules forming structures similar to micelles. This justifies the high concentration of linalool in the hydrophobic cavity when the complexes were obtained by using US, while in the case of MWI more molecules remained outside the cavity forming complexes outside of the hydrophobic cavity, favoring the linalool losses in the atomization process, thus decreasing the efficiency [41].

Therefore, although by means of MWI a greater increase in the solubility of linalool was obtained, it was observed that the complexes obtained by US contained higher concentration of linalool, since a better performance and encapsulation efficiency is obtained.

#### Stability of Solid Complexes

**Figure 5:**
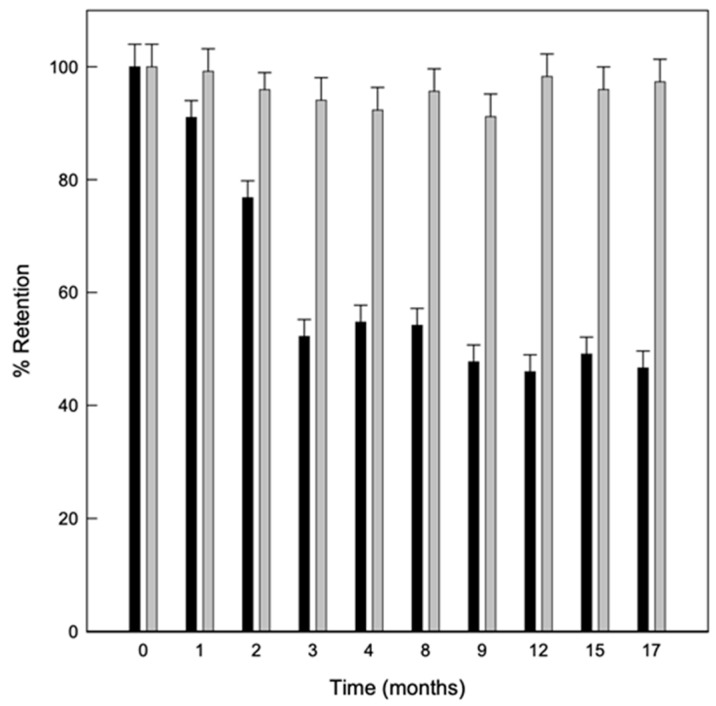
Evolution of linalool retained in solid complexes stored for 17 months at 8 °C (grey bars) and 25 °C (black bars) Values represent means of triplicate determination.

The stability of the solid complexes was also studied, evaluating the linalool content in the powder with the storage time at different temperatures. As shown in Figure 5 the powder obtained by the US maintained a greater amount of linalool retained in the samples stored at 8 °C. This was probably because at lower temperatures, there is also a lower humidity relative (RH) and therefore, fewer water molecules to compete with linalool for the hydrophobic cavity of the CDs. In fact, it has been previously described that HP-β-CDs have a higher affinity for water molecules as RH increases due to the amount of hydroxyl groups in the CDs surface, which enables the release of the complexed compound [42].

### 2.3. Characterization of Linalool-HP-β-CDs Inclusion Complexes

#### 2.3.1. ^1^H and 2D NMR Spectroscopy

To confirm the formation of the linalool-HP-β-CDs inclusion complexes, the interaction of both molecules was studied using the NMR technique, since it offers evidence of the inclusion of the host molecule inside the CDs [43]. Thus, the results obtained after subjecting the complexes to ^1^H-NMR confirmed that the stoichiometry was 1:1. 

Table 2 shows the chemical shift values of linalool and HP-β-CDs, both in their free state and complexed in methanol-d_4_ solution, as well as the differences between the signals of the free and complexed molecules. The change that occurs in the chemical shifts of the free molecule with respect to the complex, verify the formation of the inclusion complex.

**Figure 6:**
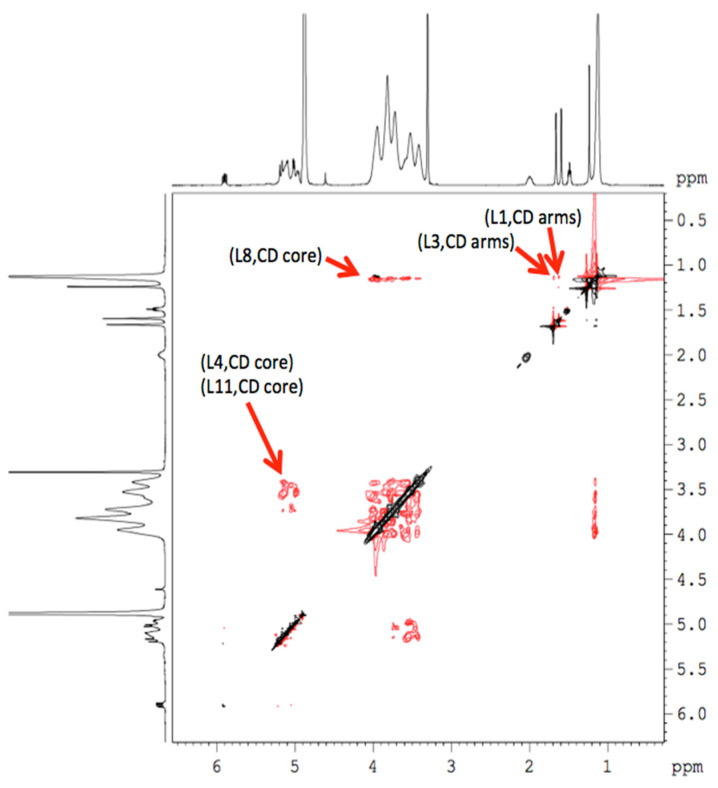
2D NMR ROESY spectrum of the linalool-HP-β-CDs complexes in methanol-d_4_.

Once the chemical shifts were obtained, a study was performed using two-dimensional (2D) NMR spectroscopy, since it provides important information on the spatial proximity between the host (HP-β-CDs) and linalool, through observation of cross correlations. This technique presents the so-called Overhauser effect (NOE) that originates from the interaction between two protons close in space, being able to observe this interaction in the nuclear Overhauser effect spectroscopy (NOESY) or rotating-frame nuclear Overhauser effect spectroscopy (ROESY) spectra, considering that the presence of crossed peaks (Figure 6), between the protons of two species in the NOE spectrum, confirms the existence of a spatial contact at 0.4 nm [9].

In order to obtain more conformational information, the 2D ROESY of the inclusion complex was obtained (Figure 6), showing an appreciable correlation (spatial proximity) of the -OH proton of linalool with the H-5 protons of HP-β-CDs. Similar interactions appeared between L4, L8 and L11 protons of linalool with the H1, H2, H3 and H4 protons of HP-β-CDs. These results confirm that linalool was included in the pocket of HP-β-CDs.

#### 2.3.2. Molecular Docking

**Figure 7:**
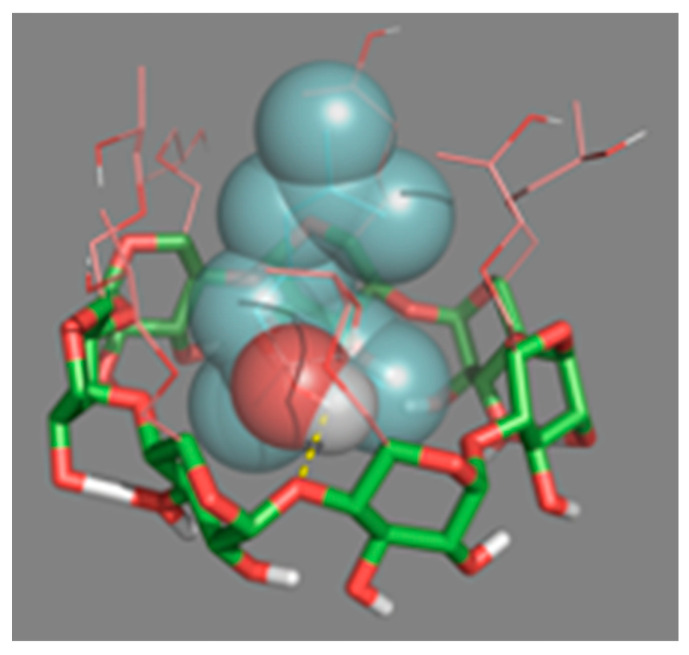
Three-dimensional perspective of the linalool-HP-β-CDs complexes obtained by Docking.

In order to understand how HP-β-CDs interact with linalool once complexed, molecular coupling simulations (docking) were performed (Figure 7). This technique showed the possible three-dimensional interaction between the HP-β-CDs atoms with linalool. It was confirmed that the hydrogen of the hydroxyl group of linalool (oxygen in red and hydrogen in gray), interacts with the H5 hydrogen atoms of HP-β-CDs. In fact, the simulation showed how the oxygen from the hydroxyl group of the CDs (red arm), interacts with linalool by forming hydrogen bonds (dashed yellow line), with the H of the –OH group (Figure 7), interacting the L4, L8 and L11 hydrogens of linalool with the hydrogen atoms H1, H2, H3 and H4 of HP-β-CDs. The simulation results agree with the data obtained by ^1^H-NMR and 2D-ROESY spectroscopy (Figure 6). Furthermore, as shown in the spherical conformation of the molecules (Figure 7), specific molecular sites in linalool are strongly attached to the hydrophobic sites in HP-β-CDs.

#### 2.3.3. Differential Scanning Calorimetry (DSC) and Thermogravimetric Analysis (TG)

**Figure 8:**
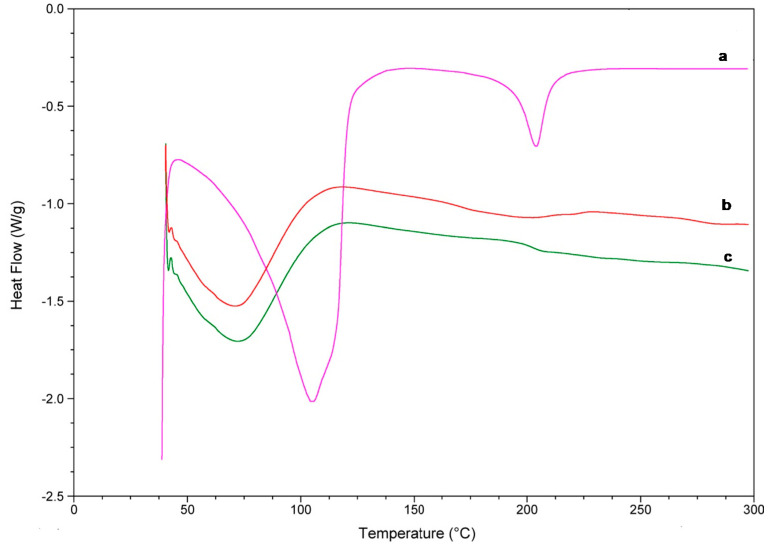
DSC curves of linalool (**a**), linalool-HP-β-CDs complexes by US (**b**), HP-β-CDs (**c**).

**Figure 9:**
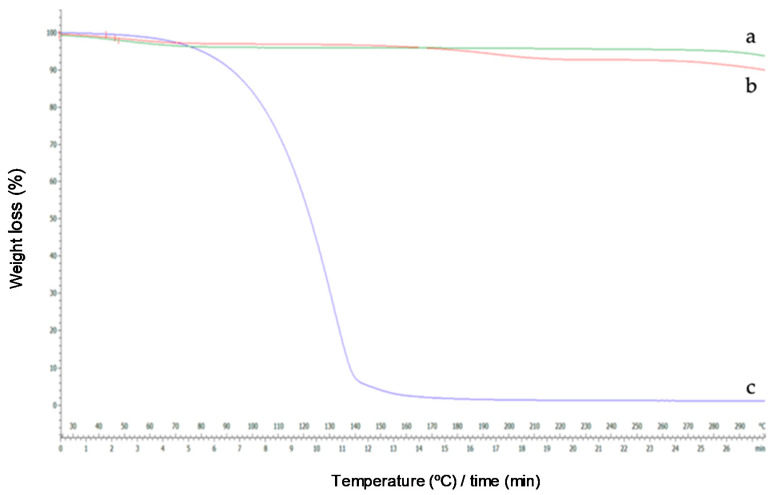
Thermograms of HP-β-CDs (**a**), linalool-HP-β-CDs complexes by US (**b**), linalool (**c**).

DSC and TG are commonly used to determine the physical properties of a substance. Through this study, the DSC thermograms of the complexes and the isolated reactants were obtained and shown in Figure 8 and Figure 9. Linalool DSC curve presented two endothermic band, one close to 100 °C and other near 210 °C, which corresponds to the oxidation and transition from a liquid to a vapour phase, respectively (Figure 8a).

For HP-β-CDs, due to its amorphous nature, a broad endothermic peak was observed at approximately 70 °C (Figure 8c) associated with the loss of water molecules. Furthermore, a small variation appears at 210 °C due to the transformation of the molecule [44,45], and a small decrease in the curve around 300 °C, probably due to the breakdown of HP-β-CDs. However, when linalool was complexed inside HP-β-CDs, an evident reduction of these signals occurred, suggesting a process of water exclusion during complex formation (Figure 8b).

The DSC curve of the inclusion complex formed by linalool-HP-β-CDs (Figure 8b) did not show the characteristic endothermic peaks of linalool (Figure 8a), indicating that the monoterpene was protected of heat treatment due to the formation of the inclusion complex with HP-β-CDs [46]. Similar results to those were described previously for linalool with β-CDs [47].

Once the results obtained by DSC were justified, the derivatives of the thermogravimetric analysis were interpreted. As can be seen in Figure 9a, the TG curve of HP-β-CDs showed a weight loss of 5%. However, the curve of the complex formed by linalool and HP-β-CDs showed a weight loss of 2.58%, because in the complex there was lower water molecules inside of the CDs cavity, since they have been displaced by linalool. In addition, a full CDs (linalool and HP-β-CDs) would be denser than an empty CDs (HP-β-CDs). Similar results were described by Fernandes et al. (2004), for linalool complexes with β-CDs [48].

#### 2.3.4. Fourier Transform Infrared Spectroscopy (FTIR)

**Figure 10:**
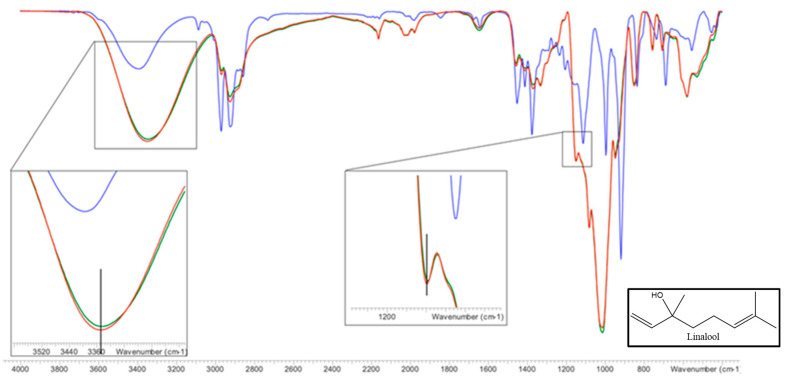
FTIR spectrum of HP-β-CDs (green line), linalool-HP-β-CDs complexes by US (red line), linalool (blue line). The vertical lines indicate the maximum of the HP-β-CDs curve. Inset: linalool chemical structure.

FTIR is a technique commonly used to confirm the formation of inclusion complexes [49]. For this reason, it was used to demonstrate the complexes formation between linalool and HP-β-CDs. Complexes and isolated molecules infrared spectra were shown in Figure 10.

Paying attention to the infrared (IR) spectrum of HP-β-CDs (Figure 10), numerous peaks appeared in the wavelength range (λ) between 600 and 3500 cm^−1^, being the most representative the following: 3342 cm^−1^ (-OH, tension vibrations); 2923 cm^−1^ (-CH, tension vibrations); 1643 cm^−1^ (-OH bending vibrations); 1157 cm^−1^ (-CO, bending vibration); 1012 cm^−1^ (-COC, stretching vibrations); 850 cm^−1^ (α-glycosidic type bond); 2967 cm^−1^ (symmetrical anti-vibration of methyl groups); 1375 cm^−1^ (methyl flex vibration).

Respect to the linalool IR spectrum (Figure 10), stretching bands associated with the -OH bonds appeared at λ = 3396 cm^−1^, out-of-plane flexion bands of -OH at λ = 1457 cm^−1^, and two bands of symmetric and asymmetric stretching of the methyl to λ = 2968 and λ = 2923 cm^−1^, respectively. A tension band of the -CO group appears at λ = 917 cm^−1^. On the other hand, the tension bands of the allyl group (C=C) are reflected in a peak that appears at λ = 1644 cm^−1^. With respect to methyl (-CH_3_) substituents appear at λ = 1375 cm^−1^. As shown in Figure 10, the spectra of HP-β-CDs and the complexes formed by linalool and HP-β-CDs were practically the same, except for some bands. The first of these is the -OH group tension band that was at 3342 cm^−1^ and moved to 3351 cm^−1^, this change occurred due to the intermolecular hydrogen bridge formed between linalool and HP-β-CDs. Another band that was modified is the one that corresponds to the -CO tension vibration zone, which appeared at 1147 cm^−1^ and moved at 1149 cm^−1^ in the complex, indicating that various interactions occur between the CDs and linalool. The characteristic band of the hydroxypropyl group of CDs did not undergo any change due to complexation, indicating that this group does not interact with linalool, confirming that the molecule is inside HP-β-CDs.

## 3. Materials and Methods

### 3.1. Materials

#### Reagents and Standards

Linalool (CAS Number 78-70-6) was purchased from Sigma (Madrid, Spain). The native α-CDs (CAS Number 10016-20-3) and β-CDs (CAS Number 7585-39-9), and the modified HP-β-CDs (CAS Number 128446-35-5), with an average degree of substitution of 0.5–1.3 units of 2-hydroxypropyl (C_3_H_7_O) for each glucose unit, were supplied by AraChem (Eindhoven, The Netherlands). The rest of the chemical reagents used were of analytical grade.

### 3.2. Preparation of Inalool-Cyclodextrin Inclusion Complexes

#### 3.2.1. Solubility Studies

The study of linalool solubility in the presence of CDs was carried out according to the method described by Higuchi and Connors in 1965 with slight modifications [50]. CDs were dissolved in buffered solutions at different pHs: 3.5, 5.5 (100 mmol L^−1^ sodium acetate buffer), 6.5, 7.0 (100 mmol L^−1^ sodium phosphate buffer) and 8.5 (100 mmol L^−1^ sodium borate buffer). The α- and HP-β-CDs were prepared up to 100 mmol L^−1^. However, β-CDs were prepared up to 15 mmol L^−1^, since this is their limit of aqueous solubility.

A saturating concentration of linalool was added to each CDs solution and they were kept in an ultrasound bath for 60 min in the dark, at 25 °C, until equilibrium was reached. Subsequently, the solutions were filtered through a 0.45 µm nylon membrane filter (Chromafil, Macherey-Nagel, Düren, Germany) to remove excess linalool.

Once filtered, the solutions were diluted with ethanol until reach ethanol 80% (solution:ethanol, 20:80, *v:v*) to quantify by gas chromatography-mass spectrometry (GC-MS) the linalool concentration in each solution. Phase solubility diagrams were made in triplicate.

#### 3.2.2. Complexation Process by Using Microwave Irradiation (MWI)

The encapsulation of linalool with HP-β-CDs by using MWI was carried out following the method described by Hernández-Sánchez et al. in 2017 [32]. Aqueous solutions of HP-β-CDs (100 mL, 0–100 mmol L^−1^) were irradiated with microwave (LG Grill Wavedom, LG Electronics Las Rozas, Spain), at 700 W for 30 s, at 10 s intervals, until reaching a temperature of 70 °C in the solution. Later on, linalool was added to each aqueous solution, in a molar ratio of 1:1 and then, were irradiated again for 30 s at 10 s intervals, until reaching again 70 °C. Subsequently, the samples were shaken and kept for 12 h in dark sealed vials at 25 °C, before dividing them into two groups. After that, the first group, named 24 h MWI, was filtered using 0.45 µm nylon membrane filter (Chromafil, Macherey-Nagel). The second group, named 48 h MWI, were irradiated again, until reaching 70 °C, and maintain 12 h in the dark before they were filtered through a 0.45 µm nylon filter to remove linalool excess. Once filtered, both groups of samples were diluted with ethanol (20:80, *v:v*) until the GC-MS quantification of the linalool. All measurements were made in triplicate.

#### 3.2.3. Quantification of Linalool by GC-MS Analysis

The quantification of linalool was carried out by using a Shimadzu GC-QP 2010 (Kyoto, Japan) gas chromatographer combined with a mass spectrometer. Helium was used as carrier gas, at a flow rate of 0.5 mL min^−1^. A ω-WAX 250 fused silica supelco column (30 m × 0.25 mm × 0.25 µm thickness), was used. The temperature conditions were as follows: initial temperature at 70 °C, raised to 160 °C at 4 °C min^−1^, raised to 280 °C at 30 °C min^−1^, and maintained finally at 280 °C for 6 min. Injector temperature was 250 °C and injector mode was Split 1:20.

The peak area of each sample was used for linalool quantification (mmol L^−1^), by interpolating in the calibration curve obtained using a standard of linalool, defined by equation: Area = 2,223,720 − 2.50 × 10^4^ [linalool (mmol L^−1^)] and (R^2^ = 0.9989) for linalool concentration from 0.0 to 0.5 mmol L^−1^.

#### 3.2.4. Complexation Constant (K_C_) and Complexation Efficiency (CE) Calculation

*Kc* between linalool and *CDs* was calculated from the slope of the phase solubility profile and the solubility of linalool in aqueous solution (*S*_0_) by using Equation (1) [50]:

(1) $$Kc\;\left(Lmol^{-1}\right)=\frac{slope}{S_{0}\left(1-slope\right)}$$

*CE* is the ratio between dissolved complex and free *CDs* concentration. It is independent of *S*_0_, and was calculated by using Equation (2) [51]:

(2) $$CE=\frac{\left[disolved-complex\right]}{\left[CDs\right]}=S_{0}*K_{c}$$

The molar ratio (MR) *linalool*:*CDs*, was calculated using *CE* values with Equation (3) [51].

(3) $$linalool:CDs=1:\left(1+\frac{1}{CE}\right)$$

### 3.3. Preparation of Linalool-HP-β-Cyclodextrin Inclusion Complexes by Spray Dryer

The equipment used was Buchi B-290 (Flawil, Switzerland). The variables used by the device were the following: inlet and outlet air temperature of 170 and 68 °C, 35 m^3^ h^−1^ for inlet air flow, 5 mL min^−1^ for pump flow and 360 L h^−1^ for compressed air flow. The recovered powder was stored in an airtight glass container at 25 and 4 °C.

The drying process yield was calculated using Equation (4):

(4) $$Drying\;process\;yield=\frac{dehydrated\;complexes\;obtained\;\left(g\right)}{total\;solids\;in\;solution\;\left(kg\right)}$$

The encapsulation yield was calculated using Equation (5):

(5) $$Linanool\;yield=\frac{total\;linalool\;in\;dehydrated\;complexes\;\left(g\right)}{total\;linaool\;in\;disolved\;complexes\;\left(kg\right)}$$

To quantify the linalool present in the solid complexes, they were solved in water (complex:water, 1:1, *w*:*v*). Solutions were then filtered using 0.45 µm nylon membrane filter (Chromafil, Macherey-Nagel). Subsequently, the dissolved filtered complexes were diluted with ethanol (20:80, *v*:*v*). The quantification of the linalool content in each solution was carried out by GC-MS in triplicate. Finally, the stability of the dehydrated HP-β-CDs-linalool complexes at 4 °C and 25 °C was measured for 17 months. All samples were analyzed in triplicate.

#### Field Emission Scanning Electron Microscope (FESEM) Images

The solid complexes were examined by field emission scanning electron microscopy (FESEM) using a MERLIN™ VP COMPACT system (Carl Zeiss Microscopy SL, Kelsterbach, Germany). The microscopy images were taken using a SE2 detector under an accelerating voltage of 1 kV.

### 3.4. Characterization of Linalool-HP-β-CDs Inclusion Complexes

#### 3.4.1. ^1^H and 2D NMR Spectroscopy

^1^H-NMR spectra of linalool, CDs, and the inclusion complexes (dissolved in D_2_O) were recorded on an Avance 600 MHz spectrometer (Bruker, Karlsruhe, Germany) at 25 °C. Chemical shifts given in parts per million (ppm), are relative to a tetramethyl silane internal standard (δ = 0.0), and NMR data were processed with MestReNova software (6.0.2–5475 version). Two-dimensional rotational frame nuclear Overhauser effect spectroscopy (2D ROESY) spectra using the standard Bruker pulse program roesygpph were acquired at 32 scans, an acquisition time of 0.150 s and a pulse delay of 2.3 s.

#### 3.4.2. Molecular Docking

In this study the molecular structures for linalool and CDs were manually constructed using AutoDockTools together with the structural information derived from experimental data [52]. The β-CDs structure was extracted from the protein structure of the Protein Data Bank (PDB) with the code 3CGT. The structure of the HP-β-CDs model was constructed by adding hydroxypropyl groups to the 𝛽-CDs model. Molecular coupling calculations were carried out using predetermined parameters [53]. The hydroxypropyl groups of HP-β-CDs were considered explicitly flexible during coupling simulations. Graphical representations of the coupling results were prepared using PyMOL (Molecular Graphics System, version 1.3, Schrödinger, LLC).

#### 3.4.3. Differential Scanning Calorimetry (DSC) and Thermogravimetric Analysis (TG)

For thermal analyses, 4–5 mg of linalool, HP-β-CDs, or complexes were weighed, to the nearest 0.1 mg in aluminum capsules. They were analyzed by differential scanning calorimetry (DSC) using a Mettler DSC Q100 analyzer (TA Instruments, Cerdanyola del Valles, Spain), and by thermogravimetry with a Hi Res TAG 2950 thermogravimetric analysis equipment (TA Instruments). The scanning temperature range was from 25 °C to 300 °C, with a scanning temperature variation of 10 °C min^−1^, using nitrogen as the carrier gas. The thermal stability of the respective components was shown using first derivative graphs (DTG) of weight (%) versus temperature (°C).

#### 3.4.4. Fourier Transform Infrared Spectroscopy (FTIR)

To determine the structural changes produced by the linalool complexation process with HP-β-CDs, the FTIR spectra were analyzed. The device used was a Varian FTIR 670 spectrophotometer (Agilent Tech, Amstelveen, The Netherlands) in combination with an accessory to analyze the attenuated total reflectance (ATR) with a wave number resolution of 0.10 cm^−1^ in the range of 250–4000 cm^−1^. A minimum of 32 scans with a resolution of 4 cm^−1^ were averaged over the above ranges.

## 4. Conclusions

This study demonstrated that linalool could be complexed with different types of native and modified CDs, increasing its aqueous by means the formation of 1:1 complexes.

The inclusion complexes of linalool and CDs depend on the pH of the reaction medium, obtaining better Kc values of at a neutral pH. The highest Kc values were obtained with HP-𝛽-CDs, followed by 𝛽-CDs, and 𝛼-CDs. Attending the results obtained of Kc values, encapsulation efficiency and stability of solid complexes, the US method is the most adequate for preparing linalool solid complexes. The results obtained by NMR and 2D-ROESY, in conjunction with molecular docking report the most probable conformation of linalool after complexation with HP-𝛽-CDs. In addition, the analyses carried out by TG, DSC, and FTIR on the free and complexed molecule, confirm that the complex formation between linalool and HP-𝛽-CDs was effective.

## Figures and Tables

**Table 1:** Recovery aqueous solubility (S_0_), complexation constant (Kc), complexation efficiency (CE) and molar ratio of linalool for α-, β- and HP-β-CDs at different pH values. Standard deviation of triplicate diagrams.

Cyclodextrin	pH	S_0_ (mmol L^−1^)	K_C_ (L mol^−1^)	CE	Molar Ratio linalool:CDs
α-CDs	3.5	9.34 ± 0.20	17 ± 6	0.159	1:7
	5.5	9.05 ± 0.16	25 ± 4	0.226	1:5
	6.5	8.16 ± 0.11	121 ± 9	0.987	1:2
	7.0	7.37 ± 0.03	148 ± 12	1.090	1:2
	8.5	9.33 ± 0.13	105 ± 8	0.979	1:2
β-CDs	3.5	9.34 ± 0.20	28 ± 4	0.261	1:5
	5.5	9.05 ± 0.16	74 ± 5	0.670	1:2
	6.5	8.16 ± 0.11	91 ± 4	0.743	1:2
	7.0	7.37 ± 0.03	241 ± 26	1.776	1:2
	8.5	9.33 ± 0.13	16 ± 5	0.149	1:7
HP-β-CDs	3.5	9.34 ± 0.20	44 ± 6	0.411	1:3
	5.5	9.05 ± 0.16	49 ± 7	0.443	1:3
	6.5	8.16 ± 0.11	225 ± 14	1.836	1:1
	7.0	7.37 ± 0.03	921 ± 21	6.788	1:1
	8.5	9.33 ± 0.13	559 ± 17	5.215	1:1

**Table 2:** Chemical shift of linalool and HP-β-CD both in its free form and complexed in methanol-d_4_.

	H-Atom	δ ppm^−1^ (Free)	δ ppm^−1^ (Complexed)	Δδ ppm^−1^ (Complexed-Free)
Linalool	H-C (3)	6.566	6.577	−0.011
	H-C (4)	6.619	6.611	0.008
	H-C (6)	6.917	6.929	−0.012
	H-C (2′)	2.740	2.758	−0.018
	H-C (5′)	2.126	2.124	0.002
	H-C (2′’)	1.176	1.195	−0.019
HP-𝛽-CDs	H-C (1)	5.074	5.121	−0.047
	H-C (2)	3.723	3.730	−0.007
	H-C (3)	3.947	3.952	−0.005
	H-C (4)	3.418	3.417	0.001
	H-C (5)	3.534	3.533	0.001
	H-C (6)	3.821	3.815	0.006
	H-C (9)	1.126	1.125	0.001
